# Supplement to Semi-Annual Report of Medical and Surgical Cases at the Buffalo Hospital of the Sisters of Charity

**Published:** 1849-11

**Authors:** 


*Suplement to semi-annual report of medical and surgical cases at the Buffalo Hospital of the Sisters of Charity.*—The September number of this Journal contained a brief report of the medical and surgical cases treated at the Buffalo Hospital of the Sisters of Charity during the second half year of the existence of the institution. We should gladly have made a more comprehensive report, including other statistics than those relating to the number of cases treated at the hospital, the diseases, and the ratio of mortality. We were prevented from so doing, by want of leisure, and, also, of space, in that number, to appropriate to the report; but we design at some future time, to supply facts interesting to the medical reader, such as the nativity, civil condition, age, sex, etc., of the patients received. The records of cases at the institution embrace all these points.

We purpose, now, to submit a financial statement of the affairs of the hospital for the first, and second semi-annual periods of its existence. This may perhaps possess some interest in connection with the considerations presented in an article contained in our last number, on the subject of Hospitals. But, for the profession in the immediate vicinity of this place, the statement may be interesting for other reasons. Strange as it may seem, to the major portion of our readers, some of the citizens of the place manifest hostility to the Hospital. This feeling of opposition, or prejudice, for the most part, is, professedly, excited by its denominational character, in a religious aspect. It is a Catholic Institution. In other words, it originated with the Rt. Rev. Dr. Timon, of this city, who has contributed, as we know, from his private resources, and from the proceeds of his labors in behalf of the Institution, several thousand dollars toward its establishment and support. Moreover, it is under the management of the Sisters of Charity, a religious order of the Roman Catholic Church. But it is a Catholic institution, in a denominational sense, only in so far as its creation, and maintenance, are, in a great measure, due to that body of christians. In so far as the sick are concerned, it is catholic, in a sense of that term to which none will take exceptions. It is open to all, without reference to religious faith, more than to country, sex, or color. No questions are asked as to theological tenets, and no distinctions are made based upon this, or any other personal consideration, except the urgency of the claims for relief. Every facility is offered for such spiritual consolation as any patient shall desire; and, with a fastidiousness which some may think is carried to an undue degree, religious observances of the Catholic Church are not offered, unsolicited, even to those who have, professedly, no religious associations, or predilections. We are led to make these statements, because we frequently have inquiries addressed to us respecting the points referred to.

Such being the unexceptionable and liberal principles with which the Institution is conducted, what ground for opposition to it can possibly exist in the minds of any person, except that it may redound to the credit of the religious denomination from which it has emanated? As a protestant, we submit, that the only proper answer to a person offering the latter objection is the following:—if you would desire to diminish the supposed advantages thus reflected upon that denomination by the benevolence of such an institution, emulate and excel in similar good works, but do not strive to withhold from the suffering sick the relief which it may afford, merely to gratify, what the exercise of christian charity should lead you to suspect may be, merely prejudice.

But we did not intend to be betrayed into any discussion of this kind, which, obviously, is out of place in the pages of a medical journal. It was necessary to allude to the existence of a feeling of bitterness in some portion of the community, in order to explain the reason, in part, for the statement of financial facts which will follow, and which has been obligingly furnished us for publication. They are submitted to meet inquiries which have been addressed to us, not only by members of the profession here, but by correspondents in other places.

The regulations of the Hospital provide for the payment of a small weekly sum by each patient, or his friends, to go to the support of the Institution. This sum, for charity patients, is $I,50 per week. The sum was fixed as low as possible, and, as it has proved, much too low to afford anything like a revenue adequate to cover the expenses for the support of the Institution. The greater number of charity patients have paid only for a short portion of time they have remained at the Hospital, and the majority, indeed, have been received without any charge. The State has made some appropriation for the support of patients at the Hospital, no portion of which has, as yet, been received.

*Financial Statement.*—The expenses of the Buffalo Hospital of the Sisters of Charity, for the relief of patients, from Sep. 1st, 1848, to March 1st, 1849, the number of patients, relieved during that period, being 121, amount to ........................................ $927,20.

| | |
|---|---|
| During the period mentioned, there were received from patients, .................................... | $263,75. |
| Amount of deficit furnished by charity, ............... | $663,45. |
| Amount of expenses for second six months, March 1, to Sept. 1, 1849, ............................. | $1,775,00. |
| Amount received from patients during same period,..... | $533,30. |
| Amount of deficit furnished by charity,............... | $1,241,70. |

It is to be observed that a considerable portion of the aggregate amounts received from patients, is paid by those who take private rooms at the Hospital, and are generally abundantly able to meet the expenses, which are $4,00 per week, exclusive of medical attendance. These persons are not, in the ordinary sense of the term, *charity patients.*

The above total amounts of expenses include only what has been expended in supporting patients at the Institution. They do not embrace very considerable outlays for stoves, bedsteads, beds and bedding, and furniture of every description. Nothing, it may be remarked, required for all possible comfort to the sick, in so far as relates to such articles, is now wanting. The purchase of the building, is of course, not included. In July, 1849, an addition, costing about $1,600,00, in the rear of the building, was completed, affording additional rooms for the sick, and, in each story, bathing rooms and waterclosets, supplied with water from tanks above.

A new additional building, costing about $5000, is now under roof, and far advanced toward completion. It will afford still greater facilities for administering comfort to the sick, affording room, also, for about double the number that the present building will accommodate. The completion of this new building will require a further expenditure of at least $2000, for necessary additional furniture, bedding, etc.

